# Female Serum HER2 Expression: Its Association With Metabolic Syndrome and Treatment Drug Response

**DOI:** 10.1155/ije/1910633

**Published:** 2026-01-31

**Authors:** Ruiwen Hu, Xiaodie He, Nan Gao, Ting He, Li Li, Tianwei Gu, Xin Gu, Cheng Ji

**Affiliations:** ^1^ Department of Pharmacy, Nanjing Drum Tower Hospital, China Pharmaceutical University, Nanjing, China, cpu.edu.cn; ^2^ Jiangsu Institute for Drug Control, Nanjing, China; ^3^ Department of Pharmacy, Nanjing Drum Tower Hospital, Clinical College of Nanjing University of Chinese Medicine, Nanjing, China, nju.edu.cn; ^4^ Department of Pharmacy, Nanjing Drum Tower Hospital, The Affiliated Hospital of Nanjing University Medical School, Nanjing, China, nju.edu.cn; ^5^ Department of Endocrinology, Endocrine and Metabolic Disease Medical Center, Nanjing Drum Tower Hospital, Affiliated Hospital of Medical School, Nanjing University, Nanjing, China, nju.edu.cn; ^6^ Department of Medical Affairs Department, Nanjing Drum Tower Hospital, The Affiliated Hospital of Nanjing University Medical School, Nanjing, China, nju.edu.cn

**Keywords:** antihypertensive agents, HER2, human epidermal growth factor receptor 2, hypolipidemic agents, metabolic syndrome, women

## Abstract

**Objective:**

To investigate the association between serum human epidermal growth factor receptor 2 (HER2) levels and metabolic syndrome (MS) in women and to explore the relationship between HER2 and the use of commonly prescribed metabolic medications.

**Methods:**

A total of 532 women who visited Nanjing Drum Tower Hospital between January 2021 and January 2023 were enrolled. Participants were classified into a non‐MS group (*n* = 278) and an MS group (*n* = 254) according to the diagnostic criteria of the Chinese Diabetes Society (2020 edition). General characteristics and serum HER2 levels were compared between groups. Based on serum HER2 levels, participants were further categorized into quartiles (Q1 [< 7.4 ng/mL], Q2 [7.4–8.5 ng/mL], Q3 [8.6–9.9 ng/mL], and Q4 [> 9.9 ng/mL]), and clinical parameters were compared among these groups. Spearman correlation analysis was performed to examine the relationships between HER2, MS‐related indicators, and medication use. Logistic regression analysis was conducted to identify independent risk factors for MS.

**Results:**

Serum HER2 levels were significantly higher in the MS group compared with the non‐MS group (8.10 [7.10–9.10] vs. 9.25 [8.10–10.80] ng/mL, *p* < 0.001). The prevalence of MS increased progressively across HER2 quartiles: 31.30% (Q1), 36.43% (Q2), 50.00% (Q3), and 72.39% (Q4) (*p* < 0.001). Serum HER2 levels were positively correlated with body mass index (BMI), waist circumference (WC), systolic blood pressure (SBP), diastolic blood pressure (DBP), glycated hemoglobin (HbA1c), fasting plasma glucose (FPG), fasting serum insulin (FINS), fasting C‐peptide (FCP), total cholesterol (TC), triglycerides (TG), and low‐density lipoprotein cholesterol (LDL‐C) (all *p* < 0.001) and negatively correlated with age, high‐density lipoprotein cholesterol (HDL‐C), and the use of renin–angiotensin system inhibitors and statins (all *p* < 0.001). Logistic regression analysis showed that higher HER2 levels remained a significant risk factor for MS after adjustment for confounders, and compared with Q1, the risk of MS in Q4 remained significantly higher in fully adjusted models.

**Conclusion:**

The use of renin–angiotensin system‐targeting antihypertensive agents or statins was associated with significantly reduced serum HER2 levels. Increasing serum HER2 levels correlated with a higher prevalence of MS, suggesting that elevated HER2 may serve as an independent risk factor and a potential biomarker for MS in women.

## 1. Introduction

Metabolic syndrome (MS) represents a constellation of interrelated cardiometabolic risk factors, including insulin resistance, impaired glucose tolerance, abdominal obesity, dyslipidemia, and hypertension, which collectively elevate the risk for cardiovascular disease and type 2 diabetes [1, 2]. In China, the prevalence of MS among adults is alarmingly high, affecting approximately 31.1% of the population, underscoring its significance as a major public health concern [2]. The pathophysiology of MS involves complex metabolic disturbances that often necessitate polypharmacy, including antihypertensive, lipid‐lowering, and glucose‐regulating agents, to mitigate its clinical manifestations and associated complications [3–5]. Despite advances in pharmacological management, the underlying molecular mechanisms linking metabolic dysfunction to therapeutic responses remain incompletely elucidated.

Human epidermal growth factor receptor 2 (HER2), a member of the epidermal growth factor receptor family, is traditionally recognized for its role in oncogenesis, particularly in breast cancer. However, emerging evidence suggests that HER2 signaling pathways are implicated in metabolic regulation, including insulin sensitivity and lipid homeostasis [6, 7]. Preclinical studies indicate that HER2 activation can influence metabolic disorders, positioning it as a potential mediator in the development and progression of MS [7]. This intersection between oncogenic signaling and metabolic pathophysiology presents a novel avenue for research, particularly given the high prevalence of both conditions in aging populations. Recent epidemiological data further highlight the systemic impact of metabolic health, as seen in conditions like metabolic dysfunction‐associated steatotic liver disease (MASLD), which affects 30%–40% of adults globally and shares common risk factors with MS, such as obesity and type 2 diabetes.

The relationship between HER2 and metabolic parameters may be bidirectional. On one hand, metabolic abnormalities characteristic of MS could modulate HER2 expression or signaling. For instance, obesity—a core component of MS—is associated with chronic inflammation and altered adipokine secretion, which may intersect with HER2 pathways to exacerbate metabolic dysfunction [5, 8]. On the other hand, HER2 itself may act as a regulator of metabolic processes. Studies have shown that HER2 signaling can impact glucose uptake and lipid metabolism, suggesting that elevated HER2 levels might contribute to insulin resistance or dyslipidemia [7]. This potential role is further supported by findings in cardio‐oncology, where HER2‐targeted therapies are associated with metabolic side effects, including changes in lipid profiles and glucose homeostasis [9].

Beyond its biological plausibility, the clinical relevance of HER2 in MS is underscored by its interaction with commonly prescribed medications for metabolic disorders. First‐line treatments for MS include antihypertensive agents such as angiotensin receptor blockers (ARBs) and lipid‐lowering drugs like statins, which have pleiotropic effects beyond their primary indications [4, 5]. These medications not only improve cardiovascular outcomes but may also modulate inflammatory and metabolic pathways that intersect with HER2 signaling. For example, statins exhibit anti‐inflammatory properties and have been shown to influence receptor tyrosine kinase activity, including pathways involving HER family members [4, 9]. Similarly, ARBs, which target the renin–angiotensin system, may affect growth factor signaling and metabolic homeostasis, though their direct impact on HER2 remains unexplored [4].

Despite these theoretical connections, empirical research on the association between serum HER2 levels and MS is scarce. Most existing studies have focused on HER2 in the context of cancer, with limited attention to its role in metabolic diseases. Furthermore, the potential influence of MS‐related pharmacotherapies on HER2 expression has not been systematically investigated. This gap is particularly relevant given the widespread use of these drugs and their potential to confound or modify the relationship between HER2 and MS. For instance, if HER2 levels are indeed elevated in MS, it remains unclear whether this elevation is a cause or consequence of metabolic dysfunction—or whether it is influenced by concomitant medications.

Recent investigations into other biomarkers and metabolic scores highlight the complexity of MS pathophysiology and the need for novel diagnostic and prognostic tools. The metabolic score for insulin resistance (METS‐IR) has been shown to predict hypertension and other cardiometabolic outcomes, suggesting that integrative biomarkers may offer superior risk stratification compared to individual parameters [10]. Similarly, the oxidative balance score (OBS), which reflects overall oxidative stress, is inversely associated with MS risk, emphasizing the role of redox imbalance in metabolic diseases [11]. In this context, HER2 could serve as a complementary biomarker, particularly if it captures unique aspects of metabolic dysregulation not fully accounted for by conventional measures.

The interplay between HER2 and metabolic health may also have implications for sex‐specific differences in MS prevalence and presentation. Women are disproportionately affected by certain metabolic conditions, such as polycystic ovary syndrome (PCOS), which often coexists with MS and involves hormonal and metabolic disturbances [12, 13]. Interestingly, HER2 expression has been studied predominantly in female populations due to its association with breast cancer, but its role in non‐oncological contexts—especially in women with MS—remains underexplored. Given that hormonal factors can influence both HER2 signaling and metabolic parameters, further research is warranted to examine potential effect modification by sex [14].

Moreover, emerging evidence suggests that dietary and lifestyle factors may modulate HER2 expression or activity indirectly through inflammatory and metabolic pathways. For example, marine‐based omega‐3 fatty acids have been shown to improve lipid profiles and reduce inflammation, both of which are relevant to MS management [15]. Similarly, gut microbiota‐modulating diets can attenuate cardiovascular–kidney–metabolic (CKM) syndrome via inflammatory mediation, highlighting the broader context in which HER2 may operate [16]. These findings suggest that HER2 could be part of a larger network of molecular and environmental factors influencing metabolic health.

In summary, while the association between HER2 and MS is biologically plausible, direct evidence from clinical studies is lacking. Existing literature has primarily focused on HER2 in oncology, with limited exploration of its metabolic roles. Additionally, the impact of commonly used MS medications—such as ARBs and statins—on HER2 levels remains unknown, creating a significant knowledge gap. This study aims to address these uncertainties by investigating the relationship between serum HER2 levels and MS in a female population, while also examining the potential moderating effects of antihypertensive and lipid‐lowering therapies. The findings may provide new insights into the pathophysiology of MS and identify HER2 as a novel biomarker or therapeutic target, ultimately contributing to more personalized and effective management strategies for this complex syndrome.

## 2. Materials and Methods

### 2.1. Study Subject

This study included 532 female patients who visited Nanjing Drum Tower Hospital between January 2021 and January 2023. According to the diagnostic criteria revised in 2020 by the Chinese Medical Association’s Diabetes Society [17], subjects were divided into a MS group (*n* = 254) and a non‐MS group (*n* = 278). Subjects were further categorized into quartiles based on serum HER2 levels: Q1 group (131 cases, HER2 < 7.4 ng/mL), Q2 group (129 cases, HER2 7.4–8.5 ng/mL), Q3 group (138 cases, HER2 8.6–9.9 ng/mL), and Q4 group (134 cases, HER2 > 9.9 ng/mL).

Inclusion criteria included (1) age ≥ 18 years and (2) undergoing serum HER2 testing. Exclusion criteria included (1) pregnancy or lactation; (2) severe cardiac, hepatic, or renal dysfunction; (3) use of hormonal medications within the past 3 months; (4) a history of malignancy; and (5) severely incomplete data. This study was approved by the Nanjing Drum Tower Hospital Ethical Committee (Approval Number: 2021‐389‐01) and informed consent was obtained from all participants.

### 2.2. Research Methods

#### 2.2.1. Data Collection

Data were collected from the clinical records of the participants, including age, smoking status, alcohol consumption, medical history (such as hypertension, diabetes, hyperlipidemia, and cancer), and categories of treatment drugs used. Information on whether and which antidiabetic drugs were used includes insulin (INS), biguanides (BGs), glucagon‐like peptide‐1 receptor agonists (GLP‐1RAs), sodium glucose transporter 2 inhibitors (SGLT‐2Is), dipeptidyl peptidase‐4 inhibitors (DPP‐4Is), α‐glucosidase inhibitors (α‐GIs), and sulfonylureas (SUs). Antihypertensive medications are categorized as renin–angiotensin–aldosterone system inhibitors (RAASIs), referring to angiotensin‐converting enzyme inhibitors (ACEIs) and ARBs, β‐blockers, and calcium channel blockers (CCBs). Antihyperlipidemics include statins, fibrates, and bile acid sequestrants (BASs). Measurements taken included height, weight, waist circumference (WC), systolic blood pressure (SBP), diastolic blood pressure (DBP), and body mass index (BMI), calculated as weight (kg) divided by the square of height (m^2^).

All subjects fasted for at least 8 h before undergoing a 75 g oral glucose tolerance test and an insulin release test the next morning. Venous blood from the elbow was collected to measure fasting plasma glucose (FPG), 2‐h postprandial blood glucose (2hPG), fasting serum insulin (FINS), 2‐h postprandial serum insulin, fasting C‐peptide (FCP), 2‐h postprandial C‐peptide (2hCP), glycosylated hemoglobin (HbA1c), total cholesterol (TC), triglycerides (TG), high‐density lipoprotein cholesterol (HDL‐C), and low‐density lipoprotein cholesterol (LDL‐C). The serum HER2 levels in all participants were determined using a double‐antibody sandwich enzyme‐linked immunosorbent assay (ELISA). The homeostatic model assessment of insulin resistance (HOMA‐IR) was calculated as follows: HOMA‐IR = FPG (mmol/L) × FINS (μIU/mL)/22.5.

#### 2.2.2. Metabolic Component Abnormalities

Abnormalities in metabolic components are defined according to the criteria in the “Guidelines for the Prevention and Treatment of Type 2 Diabetes Mellitus in China (2020 edition)” [9], which diagnoses MS based on any of the following five criteria: (1) abdominal obesity (central obesity): WC ≥ 90 cm for men and ≥ 85 cm for women; (2) elevated blood glucose: FPG ≥ 6.1 mmol/L or 2hPG ≥ 7.8 mmol/L, or cases that have been diagnosed with diabetes and treated; (3) elevated blood pressure: blood pressure ≥ 130/85 mmHg or cases that have been diagnosed with hypertension and treated; (4) fasting TG ≥ 1.70 mmol/L; (5) fasting HDL‐C < 1.04 mmol/L. A diagnosis of MS is confirmed if three or more of these criteria are met.

### 2.3. Statistical Analysis

Statistical analyses were performed using software SPSS (Version 26.0) and R (Version 4.2.2). Continuous variables following a normal distribution are presented as mean ± standard deviation and compared between groups using independent samples *t*‐tests. Variables not normally distributed are expressed as median (P_25_, P_75_) and analyzed using the Mann–Whitney *U* test for two independent samples; multiple group comparisons were conducted using one‐way ANOVA. Categorical data are expressed as counts and percentages and analyzed using the chi‐square test and trend test. Correlations between variables were assessed using Spearman’s correlation analysis; risk factors were analyzed using logistic regression. A *p* value of < 0.05 was considered statistically significant.

### 2.4. Sample Size and Power Analysis

As this was an observational cross‐sectional study, a formal a priori sample size calculation was conducted based on the objective of accurately estimating the prevalence of MS in the study population. The required sample size for prevalence estimation was calculated using the standard formula for single‐proportion estimation:

(1) n=Z2×p×1−pd2,

where *Z* represents the *Z*‐value corresponding to a 95% confidence level (1.96), *p* is the expected prevalence of MS (0.31), and *d* is the allowable margin of error (0.05). Based on this calculation, the minimum required sample size was 329 participants. The final enrollment of 532 women exceeded this requirement, ensuring sufficient precision for prevalence estimation.

## 3. Results

### 3.1. Comparison of General Data and Serum HER2 Levels Between Non‐MS and MS Groups

Patients were categorized into an MS group (*n* = 254) and a non‐MS group (*n* = 278), with an MS detection rate of 47.74%. Compared to the non‐MS group, the MS group exhibited higher serum HER2 levels (8.10 [7.10, 9.10] vs. 9.25 [8.10, 10.80] ng/mL, *p* < 0.001). No significant differences were found in smoking, alcohol consumption, or use of lipid‐lowering drugs between the groups (*p* > 0.05). The use of antidiabetic and antihypertensive medications was significantly higher in the MS group (*p* < 0.05). The MS group had significantly higher usage of insulin, GLP‐1 receptor agonists, SGLT‐2 inhibitors, calcium channel blockers, fibrates, and bile acid sequestrants compared to the non‐MS group (*p* < 0.05), while the use of statins was significantly lower (*p* < 0.05).

Significantly higher values were observed in the MS group for WC, SBP, DBP, HbA1c, FPG, 2hPG, FINS, 2hINS, FCP, 2hCP, HOMA‐IR, TC, TG, and LDL‐C (*p* < 0.05). Age and HDL‐C were significantly lower in the MS group (*p* < 0.05), as shown in Supporting Table 1.

**FIGURE 1:**
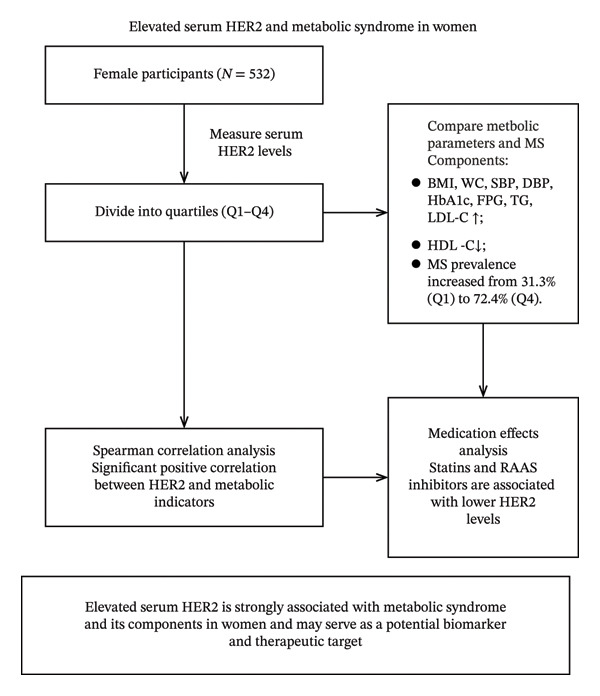
Overview of the study workflow.

The trends of MS and its components across serum HER2 quartiles are illustrated in Figure 1.

### 3.2. Comparison of General Data and MS Detection by Different Serum HER2 Levels

Participants were further categorized into quartiles according to serum HER2 levels: Q1 group (HER2 < 7.4 ng/mL), Q2 group (HER2 7.4–8.5 ng/mL), Q3 group (HER2 8.6–9.9 ng/mL), and Q4 group (HER2 > 9.9 ng/mL). No significant statistical differences were found in smoking, alcohol consumption, and the use of antidiabetic or antihypertensive medications across the four groups (*p* > 0.05). However, remarkable differences were detected in the use of lipid‐lowering drugs, angiotensin system inhibitors, statins, and fibrates, as well as age, BMI, WC, SBP, DBP, MS component scores, HbA1c, FPG, 2hPG, FINS, 2hINS, FCP, 2hCP, HOMA‐IR, TC, TG, HDL‐C, and LDL‐C (*p* < 0.05), as shown in Supporting Table 2.

**FIGURE 2:**
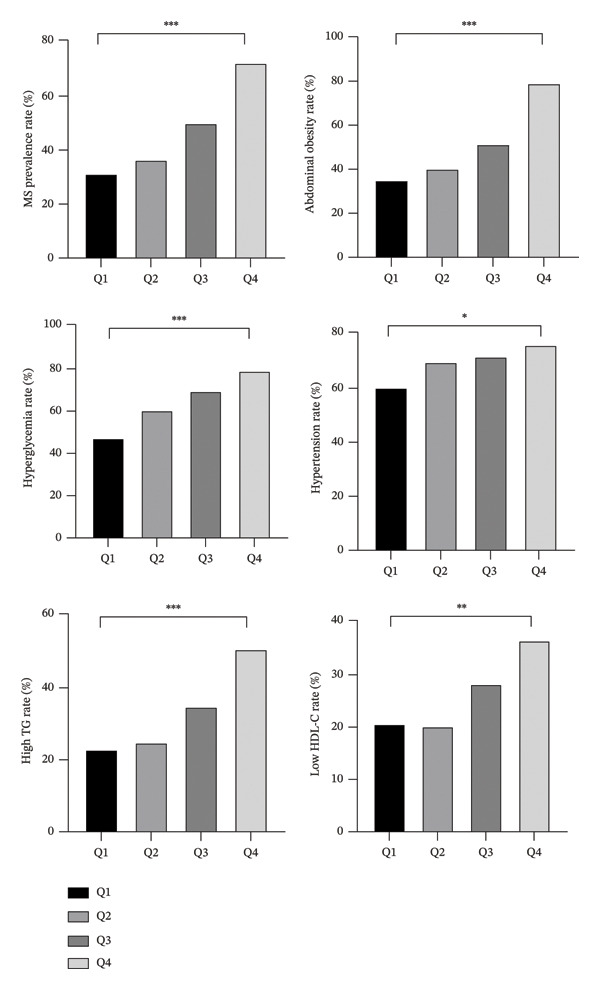
HER2 quartiles and trends in MS and its components. Note: ^∗^ *p* < 0.05; ^∗∗^ *p* < 0.01; ^∗∗∗^ *p* < 0.001; MS: metabolic syndrome; WC: waist circumference; SBP: systolic blood pressure; DBP: diastolic blood pressure; TG: triglycerides; HDL‐C: high‐density lipoprotein cholesterol.

As serum HER2 levels increased, the detection rate of MS also showed an increasing trend: 31.30% in Q1, 36.43% in Q2, 50.00% in Q3, and 72.39% in Q4 (*p* < 0.001). The rates of abdominal obesity, elevated blood glucose, hypertension, high TG, and low HDL‐C also increased with rising HER2 levels (*p* < 0.05), as depicted in Figure 2.

### 3.3. Correlation Analysis Between Serum HER2 Levels and MS‐Related Indicators

Spearman rank correlation revealed that the levels of serum HER2 were positively associated with BMI, WC, SBP, DBP, HbA1c, FPG, FINS, FCP, HOMA‐IR, TC, TG, and LDL‐C (*p* < 0.001), and negatively associated with HDL‐C (*p* < 0.001), as shown in Table 1.

**TABLE 1:** Spearman correlation analysis of HER2 levels and MS‐related indicators.

	BMI	WC	SBP	DBP	HbA1c	FPG	FINS	FCP	HOMA‐IR	TC	TG	HDL‐C	LDL‐C
*r*	0.391	0.333	0.233	0.218	0.210	0.232	0.455	0.436	0.484	0.181	0.272	−0.158	0.182
*p*	< 0.001	< 0.001	< 0.001	< 0.001	< 0.001	< 0.001	< 0.001	< 0.001	< 0.001	< 0.001	< 0.001	< 0.001	< 0.001

*Note:* HbA1c: glycosylated hemoglobin; FINS: fasting serum insulin; HOMA‐IR: homeostatic model assessment of insulin resistance; TG: triglycerides.

Abbreviations: BMI, body mass index; DBP, diastolic blood pressure; FCP, fasting C‐peptide; FPG, fasting plasma glucose; HDL‐C, high‐density lipoprotein cholesterol; LDL‐C, low‐density lipoprotein cholesterol; SBP, systolic blood pressure; TC, total cholesterol; WC, waist circumference.

### 3.4. Correlation Analysis Between Serum HER2 Levels and Treatment Medications

Spearman rank correlation indicated that serum HER2 levels showed no significant association with the overall use of antidiabetic or antihypertensive medications (*p* > 0.05). However, a negative correlation was found with the use of lipid‐lowering drugs (*r* = −0.103, *p* = 0.018). Specifically, serum HER2 levels were significantly negatively correlated with the use of angiotensin system inhibitors and statin lipid‐lowering medications (*p* < 0.001), and positively correlated with the use of fibrates and bile acid sequestrants, showing statistical significance (*p* < 0.05), as presented in Table 2.

**TABLE 2:** Spearman correlation analysis of HER2 levels and response to antidiabetic, antihypertensive, and antilipemic medications.

Category	*r*	*p*
Antidiabetic drugs	−0.018	0.680
INS	0.029	0.506
BGs	−0.077	0.076
GLP‐1RA	−0.024	0.586
SGLT‐2I	−0.050	0.250
DPP‐4I	−0.011	0.793
α‐GI	−0.072	0.099
SUs	−0.065	0.135
Antihypertensive drugs	−0.069	0.109
RAASIs	−0.164	< 0.001
β‐Blockers	−0.034	0.429
CCBs	−0.052	0.235
Antilipemic drugs	−0.103	0.018
STAT	−0.184	< 0.001
FIBs	0.129	0.003
BASs	0.101	0.020

*Note:* INS: insulin; BG: biguanide; SGLT‐2I: sodium glucose transporter 2 inhibitor; DPP‐4I: dipeptidyl peptidase‐4 inhibitor; SU: sulfonylurea; STAT: statin; FIB: fibrate.

Abbreviations: α‐GI, α‐glucosidase inhibitor; BAS, bile acid sequestrant; CCB, calcium channel blocker; GLP‐1RA, glucagon‐like peptide‐1 receptor agonists; RAASI, renin–angiotensin–aldosterone system inhibitor.

### 3.5. Multifactorial Logistic Regression Analysis of Serum HER2 and MS

A multifactorial logistic regression analysis was conducted using quartiles of serum HER2 levels as independent variables and the presence of MS as the dependent variable. With the odds ratio (OR) for MS in the Q1 group set at 1.00, the ORs (95% Confidence Interval, CI) for Q2 to Q4 were 1.26 (0.75–2.11), 2.20 (1.33–3.61), and 5.75 (3.39–9.77), respectively, as HER2 levels increased. After accounting for age, BMI, smoking history, alcohol consumption, and the use of antihypertensive and antidiabetic medications, the potential of developing MS in the Q4 group was 2.47 times higher than in the Q1 group (OR = 2.47, 95% CI: 1.28–4.78, *p* < 0.05). Further adjustment for HDL‐C indicated that the risk of MS in the Q4 group increased to 2.61 times that of the Q1 group (OR = 2.61, 95% CI: 1.21–5.64, *p* < 0.05), as shown in Table 3.

**TABLE 3:** Estimates of logistic regression models of serum HER2 and MS.

Group	Model 1		Model 2		Model 3	
	OR (95% CI)	*p*	OR (95% CI)	*p*	OR (95% CI)	*p*
Q1	1.00 (Reference)		1.00 (Reference)		1.00 (Reference)	
Q2	1.26 (0.75–2.11)	0.382	0.95 (0.50–1.83)	0.879	0.81 (0.38–1.71)	0.572
Q3	2.20 (1.33–3.61)	0.002	1.35 (0.73–2.51)	0.340	1.53 (0.74–3.14)	0.249
Q4	5.75 (3.39–9.77)	< 0.001	2.43 (1.24–4.76)	0.010	2.68 (1.23–5.87)	0.014

*Note:* Model 1: unadjusted; Model 2: adjusted for age, BMI, smoking history, alcohol consumption, antihypertensive medication, and antidiabetic medication; Model 3: adjusted for age, BMI, smoking history, alcohol consumption, antihypertensive medication, antidiabetic medication, and HDL‐C.

## 4. Discussion

MS represents a metabolic aberration distinguished by the aggregation of obesity, hypertension, hyperglycemia, and dyslipidemia. In recent years, as global economic development progresses, lifestyle changes occur, and population aging intensifies, the prevalence of MS has been rising and increasingly affecting younger populations [18]. HER2, widely recognized as a clinical tumor marker, has in recent years also been explored for its value in nontumor conditions such as insulin resistance and its related diseases [19]. However, research on the correlation between serum HER2 and MS and its components remains sparse. Therefore, this study investigates the relationship between serum HER2 and MS and its components in a female population, aiming to provide insights into the role of HER2 in metabolic disorders.

This study found that the prevalence of MS in the female population was 47.7%, with higher serum HER2 levels observed in the MS group compared to the non‐MS group. As serum HER2 levels increased, there was a corresponding rise in the detection rates of central obesity, hyperglycemia, hypertension, high TG, and low HDL‐C within MS components. After adjusting for confounding factors, multifactorial logistic regression analysis revealed that compared to the Q1 group of serum HER2 levels, the Q4 group exhibited a markedly higher risk of MS, indicating a strong positive association between HER2 levels and the risk of MS in women. A study by Qin Li et al. [20] in an elderly community population found that those in the higher quartiles of serum HER2 had higher levels of BMI, WC, SBP, DBP, FPG, 2hPG, HbA1c, HOMA‐IR, TC, TG, and LDL‐C, along with lower levels of HDL‐C. Furthermore, as the number of MS risk factors increased, serum HER2 levels also rose, significantly correlating with an increased prevalence of MS, aligning closely with the findings of this study.

This study demonstrates a positive correlation between HER2 and MS‐related indicators such as WC and BMI. The pathogenesis of MS is complex, with numerous studies identifying obesity, particularly central obesity, as a critical causative factor [21]. Emerging evidence suggests that HER2 may actively participate in adipogenesis and energy regulation, expanding its functional profile beyond oncology. Experimental studies have demonstrated that HER2 can phosphorylate fatty acid synthase (FASN), a key enzyme in lipid synthesis, thereby promoting adipocyte differentiation and lipid accumulation [22, 23]. Additionally, HER2 levels have been reported to correlate with insulin resistance in obese individuals and decrease after weight loss, indicating that HER2 expression may be modulated by metabolic status [24]. Molecular studies further indicate that HER2 plays a role in the differentiation of preadipocytes into mature adipocytes, potentially contributing to adipose tissue expansion and metabolic dysfunction [25].

Beyond obesity, circulating HER2 levels also demonstrated meaningful associations with hypertension. In this study, HER2 levels were negatively correlated with the use of antihypertensive renin–angiotensin–aldosterone system (RAAS) inhibitors, while showing a positive correlation with blood pressure values. Previous research has identified angiotensin‐converting enzyme (ACE) as a mediator of serum HER2 regulation and observed that patients using ACEIs or ARBs exhibit significantly reduced HER2 levels [9]. These findings may explain the positive correlations observed between HER2 and both SBP and DBP, suggesting that HER2 may influence vascular homeostasis and endothelial function.

The relationship between HER2 and glucose metabolism was also evident in this study, as higher HER2 levels were associated with increased prevalence of hyperglycemia and significantly elevated FPG, HbA1c, and insulin levels, consistent with longitudinal evidence reporting that individuals with elevated HER2 levels have a substantially higher risk of developing diabetes [26]. HER2 is closely linked to FASN, a molecule involved in glucose metabolic regulation [27], and FASN has been proposed as a potential biomarker of insulin resistance and type 2 diabetes [28]. These findings suggest that HER2 may influence glucose dysregulation through FASN‐dependent mechanisms, potentially amplifying metabolic deterioration.

Lipid abnormalities, a hallmark of MS, also showed significant associations with HER2. HER2 was negatively correlated with HDL‐C and statin use but positively correlated with TG, TC, LDL‐C levels, and fibrate use. Statins inhibit the rate‐limiting enzyme HMG‐CoA reductase, reducing serum lipid levels and slightly increasing HDL‐C while additionally exhibiting anticancer effects [29], which may explain their inverse association with HER2. Fibrates, by contrast, regulate the NPC1L1 transporter and enhance hepatic LDL metabolism [30], consistent with our findings. HER2 also interacts with key lipid metabolic enzymes such as PI3K and mTOR, and dietary fatty acids (omega‐3 vs. omega‐6) have been shown to modulate extracellular HER2 concentrations [31], indicating a bidirectional relationship between lipid metabolism and HER2 signaling.

The observed inverse associations between HER2 levels and the use of statins and RAAS inhibitors imply potential medication‐dependent regulation of HER2 expression, suggesting that these commonly prescribed drugs may exert indirect therapeutic effects on metabolic pathways involving HER2.

Despite these compelling findings, the mechanisms through which HER2 influences MS remain incompletely understood. HER2 may act as a central signaling hub mediating pathways related to lipid metabolism, insulin sensitivity, oxidative stress, inflammation, and mitochondrial regulation via PI3K/AKT and MAPK pathways [32–34]. Further mechanistic and interventional research is needed to clarify these processes.

Finally, given its accessibility and strong associations with multiple metabolic indicators, serum HER2 may have potential utility as a biomarker for MS. However, whether HER2 elevation precedes or results from metabolic dysfunction remains unclear, necessitating longitudinal validation.

This study has limitations. Firstly, it is a cross‐sectional study, which does not allow for the determination of causal relationships between serum HER2 levels and the risk of developing MS. Secondly, the study population primarily consists of female patients visiting the hospital, which may introduce selection bias, limiting the generalizability of the results to other specific populations. Lastly, while some common confounding factors such as age and BMI were considered, other potential confounders such as diet, exercise, and habits were not included in the analysis, which may affect the outcomes. Future research will incorporate prospective cohort designs, expanded population diversity, and mechanistic experimentation to clarify causality and evaluate metabolic effects of HER2‐targeted interventions.

## 5. Conclusion

The findings of this study show that serum HER2 levels in the female population are closely related to MS and its components, such as central obesity, hypertension, and abnormalities in glucose and lipid metabolism. The use of antihypertensive and lipid‐lowering medications also influences serum HER2 levels. As HER2 levels increase, the risk of MS significantly rises, making HER2 one of the risk factors for MS in women. This suggests a link between elevated HER2 levels and metabolic disorders. HER2 levels may be modulated by antihypertensive and lipid‐lowering medications, and HER2 could serve as a promising target for the intervention and therapy of MS.

## Author Contributions

Ruiwen Hu: methodology, writing–original draft, software, and visualization. Xiaodie He: methodology, writing–original draft, software, and visualization. Nan Gao: writing–original draft and data curation. Ting He: writing–original draft and data curation. Li Li: writing–review and editing and investigation. Tianwei Gu: methodology, writing–review and editing, and conceptualization. Xin Gu: writing–review and editing, project administration, and supervision. Cheng Ji: funding acquisition, writing–review and editing, and project administration.

## Funding

This work was supported by the Zhejiang Yangtze River Delta Health Research Fund Project (grant number: 2023CSJ‐2‐A001) and Funding for Clinical Trails from the Affiliated Drum Tower Hospital, Medical School of Nanjing University (grant number: 2021‐LCYJ‐PY‐33).

## Ethics Statement

The studies involving humans were approved by the Nanjing Drum Tower Hospital Institutional Review Board (number: 2021‐389‐01). The studies were conducted in accordance with the local legislation and institutional requirements. Written informed consent for participation in this study was provided by the participants’ legal guardians/next of kin.

## Conflicts of Interest

The authors declare no conflicts of interest.

## Supporting Information

The Supporting Information for this article can be found in the attachment “Supporting Information”.

Supporting Table 1: Comparison of Descriptive Statistics and Serum HER2 Levels between Non‐MS and MS Groups [$\overset{¯}{x}\pm s$, M (P_25_, P_75_), *n* (%)].

Supporting Table 2: Comparison of Descriptive Statistics Across Different Serum HER2 Quartiles [$\overset{¯}{x}\pm s$, M (P_25_, P_75_), *n* (%)].

## Supporting information


**Supporting Information** Additional supporting information can be found online in the Supporting Information section.

## Data Availability

The original contributions presented in the study are included in the article/Supporting Information. Further inquiries can be directed to the corresponding authors.
