# Supplementary material for: Female Serum HER2 Expression: Its Association With Metabolic Syndrome and Treatment Drug Response
Source: Int J Endocrinol. 2026 Jan 31;2026:1910633. doi: 10.1155/ije/1910633 (PMC12860420; doi:10.1155/ije/1910633)
Supplement: Supplementary file 1 — Supporting Information Additional supporting information can be found online in the Supporting Information section. [file IJE-2026-1910633-s001.docx]

Supplementary Material

**Supplementary Table 1 Comparison of Descriptive Statistics and Serum HER2 Levels between Non-MS and MS Groups[x̅±s, M(P25，P75), n(%)]**

| Characteristic  Project | Non-MS, N = 278 | MS, N = 254 | Statistic | ***P* value** |
| --- | --- | --- | --- | --- |
| Age (years) | 50.00 (35.00 , 60.00) | 40.00 (31.00 , 57.00) | Z=-3.282 | 0.001 |
| BMI（kg/m^2^) | 23.71 (21.34 , 26.49) | 33.08 (27.62 , 37.78) | Z=-13.077 | <.001 |
| Smoking（%） | 8 (2.88) | 14 (5.51) | χ²=2.323 | 0.127 |
| Drinking（%） | 3 (1.08) | 5 (1.97) | χ²=0.236 | 0.627 |
| **Antidiabetic Drugs（%）** | 84 (30.22) | 108 (42.52) | χ²=8.711 | 0.004 |
| INS（%） | 46 (16.55) | 68 (26.77) | χ²=8.242 | 0.004 |
| BGs（%） | 47 (16.91) | 49 (19.29) | χ²=0.510 | 0.475 |
| GLP-1RA（%） | 7 (2.52) | 31 (12.20) | χ²=18.777 | <.001 |
| SGLT-2I（%） | 13 (4.68) | 30(11.81) | χ²=9.094 | 0.003 |
| DPP-4I（%） | 32 (11.51) | 33 (12.99) | χ²=0.272 | 0.602 |
| α-GI（%） | 33 (11.87) | 20 (7.87) | χ²=2.363 | 0.124 |
| SUs（%） | 7 (2.52) | 6 (2.36) | χ²=0.014 | 0.907 |
| **Antihypertensive Drugs（%）** | 89 (31.01) | 122 (48.03) | χ²=14.229 | <.001 |
| RAASIs（%） | 57 (20.50) | 51 (20.08) | χ²=0.015 | 0.903 |
| β-blockers（%） | 13 (4.68) | 15 (5.91) | χ²=0.402 | 0.526 |
| CCBs（%） | 56 (20.14) | 104 (40.94) | χ²=27.308 | <.001 |
| **Antilipemic Drugs（%）** | 101 (36.33) | 92 (36.22) | χ²=0.001 | 0.979 |
| STAT（%） | 96 (34.53) | 64 (25.20) | χ²=5.501 | 0.019 |
| FIBs（%） | 6 (2.16) | 24 (9.45) | χ²=13.258 | <.001 |
| BASs（%） | 0 (0) | 7 (2.76) | χ²=7.764 | 0.005 |
| WC（cm） | 88.31 ± 14.92 | 107.67 ± 16.53 | t=-7.083 | <.001 |
| SBP（mmHg） | 124.00 (115.00 , 138.00) | 140.00 (126.25 , 151.00) | Z=-7.445 | <.001 |
| DBP（mmHg） | 77.00 (70.00 , 84.00) | 85.00 (76.25 , 98.00) | Z=-7.217 | <.001 |
| HbA1c（%） | 5.50 (5.20 , 6.00) | 6.10 (5.60 , 7.73) | Z=-7.255 | <.001 |
| FPG（mmol/L） | 4.84 (4.46 , 5.67) | 5.78 (5.00 , 7.77) | Z=-8.083 | <.001 |
| 2hPG（mmol/L） | 7.60 (6.10 , 12.00) | 10.45 (8.51 , 14.67) | Z=-7.056 | <.001 |
| FINS（μIU/ml） | 7.37 (5.01 , 12.27) | 18.80 (10.40 , 30.10) | Z=-10.025 | <.001 |
| 2hINS（μIU/ml） | 52.50 (29.15 , 90.80) | 100.00 (45.50 , 180.00) | Z=-6.020 | <.001 |
| FCP（pmol/L） | 627.00 (484.50 , 853.00) | 1097.50 (821.25 , 1481.75) | Z=-10.812 | <.001 |
| 2hCP（pmol/L） | 2652.00 (1758.00 , 3676.00) | 3727.00 (2283.00 , 4934.00) | Z=-5.257 | <.001 |
| HOMA-IR | 1.74 (1.16 , 2.99) | 5.38 (2.87 , 8.63) | Z=-11.606 | <.001 |
| TC（mmol/L） | 4.62 (3.94 , 5.26) | 4.83 (4.15 , 5.61) | Z=-2.401 | 0.016 |
| TG（mmol/L） | 1.04 (0.77 , 1.32) | 1.92 (1.45 , 2.49) | Z=-14.428 | <.001 |
| HDL-C（mmol/L） | 1.44 (1.19 , 1.75) | 1.05 (0.91 , 1.21) | Z=-12.359 | <.001 |
| LDL-C（mmol/L） | 2.60 (2.05 , 3.22) | 2.90 (2.33 , 3.42) | Z=-3.260 | 0.001 |
| HER2（ng/mL） | 8.10 (7.10 , 9.10) | 9.25 (8.10 , 10.80) | Z=-7.197 | <.001 |

**Supplementary Table 2 Comparison of Descriptive Statistics Across Different Serum HER2 Quartiles[x̅±s, M(P25，P75), n(%)]**

| Characteristic  Group | Q1  （HER2＜7.4 ng/mL） | Q2  （HER2 7.4-8.5 ng/mL） | Q3  （HER2 8.6-9.9 ng/mL） | Q4  （HER2＞9.9ng/mL） | *P* for trend |
| --- | --- | --- | --- | --- | --- |
| Age (years) | 47.00 (36.00 , 58.50) | 54.00 (40.00 , 63.00) | 47.50 (32.00 , 61.00)^b^ | 32.50 (27.00 , 50.75)^abc^ | <.001 |
| BMI（kg/m^2^) | 24.60 (21.30 , 28.89) | 25.05 (22.12 , 30.44) | 26.82 (23.90 , 34.38)^ab^ | 34.50 (28.32 , 39.36)^abc^ | <.001 |
| Smoking（%） | 4 (3.05) | 6 (4.65) | 7 (5.07) | 5 (3.73) | 0.841 |
| Drinking（%） | 2 (1.53) | 1 (0.78) | 2 (1.50) | 3 (2.24) | 0.812 |
| **Antidiabetic Drugs（%）** | 49 (37.40) | 46 (35.66) | 48(34.78) | 49 (36.57) | 0.974 |
| INS（%） | 25 (19.08) | 24 (18.60) | 36 (25.36) | 30 (22.39) | 0.497 |
| BGs（%） | 25 (19.08) | 28 (21.71) | 25 (18.12) | 18 (13.43) | 0.363 |
| GLP-1RA（%） | 14 (10.69) | 5 (3.88) | 8 (5.80) | 11 (8.21) | 0.160 |
| SGLT-2I（%） | 12 (9.16) | 10 (7.75) | 13 (9.42) | 8 (5.97) | 0.715 |
| DPP-4I（%） | 14 (10.69) | 22 (17.05) | 16 (11.59) | 13 (9.70) | 0.268 |
| α-GI（%） | 16 (12.21) | 13 (10.08) | 15 (10.87) | 9 (6.72) | 0.486 |
| SUs（%） | 4 (3.05) | 5 (3.88) | 4 (2.90) | 0 (0) | 0.188 |
| **Antihypertensive Drugs（%）** | 52 (39.69) | 60 (46.51) | 54 (39.13) | 45 (33.58) | 0.202 |
| RAASIs（%） | 30 (22.90) | 37 (28.68) | 30 (21.94) | 11 (8.21) | <.001 |
| β-blockers（%） | 10 (7.63) | 6 (4.65) | 5 (3.62) | 7 (5.22) | 0.509 |
| CCBs（%） | 38 (29.01) | 47 (36.43) | 38 (27.54) | 37 (27.61) | 0.339 |
| **Antilipemic Drugs（%）** | 46 (35.11) | 62 (46.80) | 51 (36.96) | 34 (25.37) | 0.002 |
| STAT（%） | 45 (34.35) | 54 (41.86) | 43 (31.16) | 18 (13.43) | <.001 |
| FIBs（%） | 1 (0.76) | 8 (6.20) | 8 (5.80) | 13 (9.70) | 0.018 |
| BASs（%） | 0 (0) | 0 (0) | 3 (2.17) | 4 (2.99) | 0.068 |
| WC（cm） | 94.12 ± 15.55 | 93.11 ± 16.87 | 100.42 ± 17.73 | 110.80 ± 17.95^abc^ | <.001 |
| SBP（mmHg） | 123.00 (113.00 , 140.00) | 129.00 (119.00 , 143.00)^a^ | 132.00 (123.00 , 146.00)^a^ | 138.00 (126.00 , 151.50)^abc^ | <.001 |
| DBP（mmHg） | 79.00 (68.50 , 86.00) | 79.00 (73.00 , 87.00) | 81.00 (74.00 , 91.75)^a^ | 84.50 (77.00 , 98.00)^abc^ | <.001 |
| MS Component Count | 2.00 (1.00 , 3.00) | 2.00 (1.00 , 3.00) | 2.50 (2.00 , 3.00)^ab^ | 3.00 (2.00 , 4.00)^abc^ | <.001 |
| HbA1c（%） | 5.50 (5.20 , 6.60) | 5.70 (5.40 , 6.70) | 5.80 (5.45 , .50)^a^ | 6.00 (5.60 , 7.53^)ab^ | <.001 |
| FPG（mmol/L） | 4.90 (4.36 , 5.93) | 5.05 (4.66 , 6.72) | 5.25 (4.71 , 6.97) | 5.62 (4.92 , 7.87)^abc^ | <.001 |
| 2hPG（mmol/L） | 7.70 (5.80 , 11.50) | 9.60 (7.10 , 13.80)^a^ | 9.20 (7.50 , 13.55)^a^ | 9.70 (8.10 , 14.45)^a^ | <.001 |
| FINS（μIU/ml） | 6.67 (4.87 , 11.60) | 8.75 (5.40 , 15.42) | 12.25 (7.03 , 20.80) | 23.80 (12.83 , 33.18)^abc^ | <.001 |
| 2hINS（μIU/ml） | 43.90 (18.15 , 72.55) | 67.30 (44.05 , 113.00)^a^ | 66.30 (40.70 , 132.00)^a^ | 120.50 (50.90 , 208.50)^abc^ | <.001 |
| FCP（pmol/L） | 612.00 (478.75 , 913.25) | 708.00 (535.00 , 986.50) | 867.00 (617.00 , 1121.50)^ab^ | 1312.00 (847.00 , 1624.75)^abc^ | <.001 |
| 2hCP（pmol/L） | 2456.50 (1594.50 , 3419.75) | 3040.00 (2238.00 , 4025.00)^a^ | 3142.00 (1905.00 , 4233.00)^a^ | 4025.50 (2480.50 , 5142.75)^abc^ | <.001 |
| HOMA-IR | 1.63 (1.14 , 2.67) | 1.97 (1.40 , 4.32) | 3.41 (1.69 , 5.66) | 6.81 (3.31 , 9.63)^abc^ | <.001 |
| TC（mmol/L） | 4.41 (3.87 , 5.14) | 4.67 (4.01 , 5.24) | 4.86 (4.22 , 5.50)^a^ | 4.93 (4.29 , 5.69)^ab^ | 0.003 |
| TG（mmol/L） | 1.11 (0.77 , 1.58) | 1.25 (0.96 , 1.68) | 1.43 (1.03 , 1.99) | 1.71 (1.18 , 2.26)^ab^ | <.001 |
| HDL-C（mmol/L） | 1.29 (1.09 , 1.65) | 1.22 (1.06 , 1.46) | 1.23 (1.02 , 1.54) | 1.14 (0.95 , 1.35)^abc^ | <.001 |
| LDL-C（mmol/L） | 2.45 (2.01 , 3.18) | 2.66 (2.08 , 3.21) | 2.83 (2.29 , 3.36)^a^ | 2.90 (2.40 , 3.45)^ab^ | 0.002 |

**a** compared to Q1, P < 0.05; **b** compared to Q2, P < 0.05; **c** compared to Q3, P < 0.05
